# Change in Physical Activity Patterns Through a School-Based Intervention: The Altus Health and Wellness Academy Model

**DOI:** 10.1016/j.focus.2026.100494

**Published:** 2026-03-26

**Authors:** Lindsey Strieter, Shuaijie Wang, Tanvi Bhatt, John Heybach, Ross Arena

**Affiliations:** 1 Department of Physical Therapy, College of Applied Health Sciences, University of Illinois Chicago, Chicago, Illinois; 2 Healthy Living for Pandemic Event Protection (HL – PIVOT) Network, Chicago, Illinois; 3 Altus Academy, Chicago, Illinois; 4 HealthPartners Institute, Minneapolis, Minnesota

**Keywords:** Unhealthy lifestyle behaviors, social vulnerability, sedentary behavior, school programs, physical activity

## Abstract

**Introduction:**

Physical inactivity continues to be a leading health crisis in the U.S. There are clear differences in physical inactivity prevalence across U.S. regions with unique distinctions and drivers. This study reports on improvements in self-reported physical activity in a school-based, K-8 health and wellness program in an underserved community in Chicago. In addition, the study also examines how the social vulnerability of the participants in the program compares with state and national standards. This study utilized a longitudinal cohort design.

**Methods:**

Seventy-two students participated in the Health and Wellness Academy, with a mean age of 10.43±2.18 years. Social vulnerability was higher in neighborhoods where students participating in the Health and Wellness Academy lived than Illinois overall and the national average. Among these participants, 58.3% were female, 54 of 72 (75%) were Hispanic, and the remaining 18 (25%) were Black. Each Health and Wellness Academy session had a portion of time dedicated to physical activity.

**Results:**

Results indicated that physical activity levels increased along with time points. Mixed linear model regression results further revealed a significant increase for time effects on physical activity (*p*<0.001).

**Conclusions:**

Having a consistent and sustained intervention allows youth to be exposed to a variety of physical activity options. These findings indicate that the Health and Wellness Academy’s tailored model allows for youth to find a physical activity that is meaningful and relevant, thus implementing it into their daily lives.

## HIGHLIGHTS

• Physical inactivity continues to be a health crisis in the U.S.
• There is a strong relationship between social vulnerability and physical inactivity prevalence.
• In socially vulnerable youth, physical activity levels increased with a school-based program.
• Consistent and sustained intervention allows exposure to a variety of physical activity options.

## INTRODUCTION

Physical inactivity continues to be a health crisis in the U.S.^1^^,^^2^ This modifiable health behavior is a leading driver for risk of chronic disease and poor health outcomes across the lifespan.^3^, ^4^, ^5^ The health benefits of moving more and sitting less are beyond dispute and recognized by the healthcare sector as well as multiple other stakeholders (e.g., public health, government, community, education systems, and others).^6^^,^^7^ Clearly, there is a broad recognition of the urgency of improving population-level physical inactivity patterns across the lifespan (i.e., children to the elderly).^8^

Despite evidence supporting the health yield of increasing physical activity levels, from the individual to population level, little to no progress has been made in improving the unacceptable prevalence of physical inactivity.^9^ The characterization of physical inactivity as a pandemic is well deserved.^1^ If there is any hope of addressing and reversing the U.S. physical inactivity pandemic, understanding the true drivers of unhealthy behavior choices must become a priority.

There are clear differences in physical inactivity prevalence across U.S. regions with culturally distinct identities.^10^ Recent work has demonstrated a strong relationship between social vulnerability and physical inactivity prevalence, indicating that underrepresented individuals in underserved communities are particularly susceptible to a sedentary lifestyle and the subsequent increased risk of a poor health trajectory throughout life.^11^^,^^12^ Therefore, the drivers of unhealthy lifestyle behaviors are multifactorial and complexly interact on a local level.

Ideally, unhealthy lifestyle behaviors should be addressed through a primary prevention framework to prevent the development of chronic disease risk factors before they become entrenched. However, the U.S. healthcare system largely operates within a reactive, secondary prevention model, focusing on disease treatment after onset—often at advanced stages. Given that a large-scale paradigm shift toward primary prevention within the healthcare system is unlikely in the near future, there is an urgent need for effective, scalable behavior-change strategies outside of traditional healthcare settings.

One such approach is the University of Illinois Chicago (UIC) Health and Wellness Academy (HWA), a multitiered, multicomponent, tailored health program that provides mentorship and hands-on experiences for youth in underserved communities. In addition to program delivery, HWA functions as a research hub, maintaining data to inform, evaluate, and sustain evidence-based educational services. Prior findings demonstrate improvements in nutritional intake and physical activity among participating youth, aligning with core program objectives. This study reports changes in self-reported physical activity among youth participating in the UIC HWA program within an underserved Chicago community.

## METHODS

### Study Sample

Altus Academy is a private, nonprofit school (https://altusacademy.org/) that enrolls around 80 students each academic year. All Altus students participate in HWA as an elective course; however, data are only stored and analyzed for participants who assented, and parents consented to the research. Participants for the HWA research were recruited on the basis of an announcement script describing the research and educational goals. The mean age of the cohort assessed was 10.43±2.18 years. Among these participants, 58.3% were female, 54 of 72 (75%) were Hispanic, and the remaining 18 (25%) were Black. This study was approved by the IRB at the University of Illinois at Chicago (STUDY2018-0965). All students and their parents provided consent for physical activity data collection through surveys at baseline, Year 2, Year 3, Year 4, and Year 5. Among these 72 students, 48 participants (aged 9.81±1.52 years; 52.1% female; 77% Hispanic, 23% Black) completed the baseline assessment and at least 1 follow-up assessment (Years 2–5), which were included in this study. On average, they participated in 2.6±1.5 follow-up assessments.

### Intervention

HWA set out with 2 programming objectives. First, to create a service-learning course for UIC undergraduate and graduate students where they could be immersed in experiential learning and mentorship. This required the application of didactic content in a real-world setting and interpersonal skills to motivate behavior change and promote healthy living. Second, HWA aims to create a wellness program for youth in the surrounding community, where historically underserved populations could be engaged in lessons on nutrition, physical activity, mindfulness, and other healthy living behaviors. Its program objectives are to enhance nutrition intake and increase physical activity.

The HWA is implemented for 1 hour each week for 30 weeks per school year. Because of the enrollment number of the school (number of students attending Altus), there are 2 sessions—one for sixth through eighth graders and the second for first through fifth graders. Each session contained a nutrition component and a physical activity component. The second half hour of each session was dedicated to physical activity. For age-appropriate outcomes, HWA utilized the Center for Disease Control and Prevention Physical Activity Guidelines (2^nd^ Edition) and the National Health Education Standards.^13^ Participants engaged in 3 types of physical activity: aerobic, muscle strengthening, and bone strengthening with National Health Education Standards–aligned learning objectives. Lessons focused on students being able to perform physical activity at home or during leisure time at school. Each week, families received information and correspondence regarding the weekly nutrition lesson, recipe, and physical activity lesson, thus allowing families to implement content at home.

### Measures

The Champions for Change Compendium of Surveys for Nutrition Education and Obesity Prevention Section 1.1 Network Youth Surveys^14^ is used as a self-reported measure of daily nutrition intake and weekly physical activity, developed by the California Department of Public Health.^15^ The survey was adapted from the School Physical Activity and Nutrition Project (University of Texas)^16^, ^17^, ^18^ and the Fruits and Veggies More Matters Consumption Survey (Arizona Nutrition Network)^19^^,^^20^ by the Research and Evaluation section of the *Network for a Healthy California* and funded by U.S. Department of Agriculture Supplemental Nutrition Assistance Program. This validated physical activity tool provides a user-friendly platform of tailored language and colorful images, completed in minimal time and with little assistance. All students included in the current analysis completed the assessment prior to HWA enrollment and at the end of each school year after 30 weeks of the HWA intervention.

**Figure 1:**
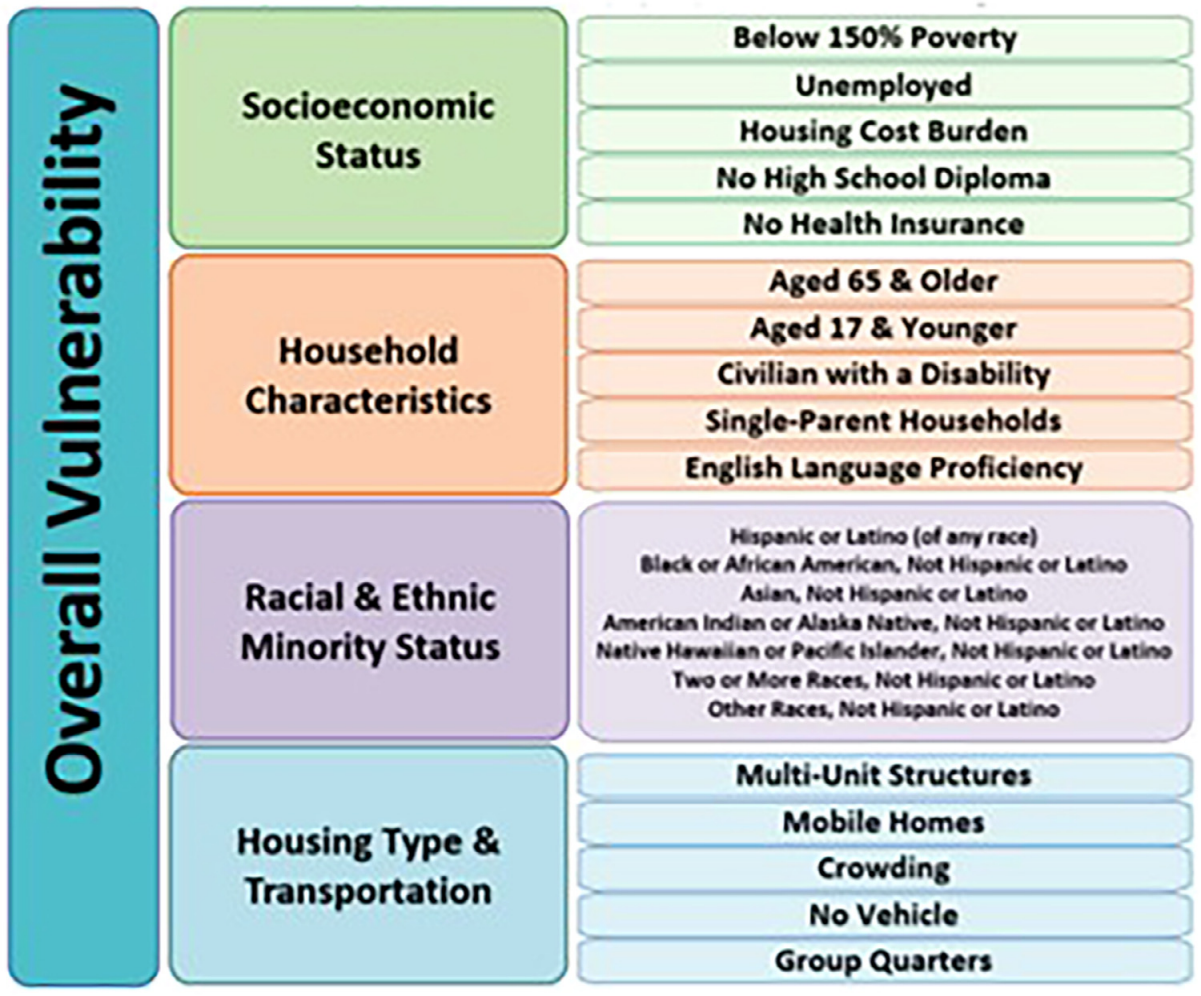
Variables included in the CDC/ATSDR Social Vulnerability Index. *Note*: Measures are derived from 5-year (2018–2022) American Community Survey data. ATSDR, Agency for Toxic Substances and Disease Registry; CDC, Center for Disease Control and Prevention.

Source: CDC SVI Documentation 2022: https://www.atsdr.cdc.gov/placeandhealth/svi/documentation/SVI_documentation_2020.html.

The 2022 Center for Disease Control and Prevention/Agency for Toxic Substances and Disease Registry Social Vulnerability Index (SVI) database was used to determine overall SVI score as well as subscore for the following: (1) average county level for the entire U.S., (2) average census-tract level for state of Illinois, and (3) average for census tracts where students participating in this study lived.^21^ The SVI seeks to capture the potential negative effects on communities due to external health stressors using 16 U.S. census variables; there are 4 SVI subthemes, and each is calculated as the sum of percentages (0–1.0) for each constituent variable (Figure 1). Overall, SVI is the sum of the 4 subthemes, and higher scores indicate greater social vulnerability. Data used to calculate the 2022 SVI were derived from 5-year American Community Survey data (2018–2022).

### Statistical Analysis

Mixed linear model regression analyzed the changes in physical activity and outdoor activity over time after HWA implementation. This method appropriately accounts for the correlation between repeated observations within individuals and accounts for missing data. When significant time effects were detected, Dunn’s test with Bonferroni Correction was used as a posthoc test for multiple comparison between different time points. Similarly, mixed linear model regression estimated the age effects on physical activity and outdoor activity at baseline (time point=0) and follow-up assessments (time points=1–4), respectively. Dunn’s test with Bonferroni correction was used when significant age effects were detected. All data analysis and statistical analysis were performed using Python, Version 3.12.10. Statistical significance was set at *p*<0.05 for all tests.

## RESULTS

**Figure 2:**
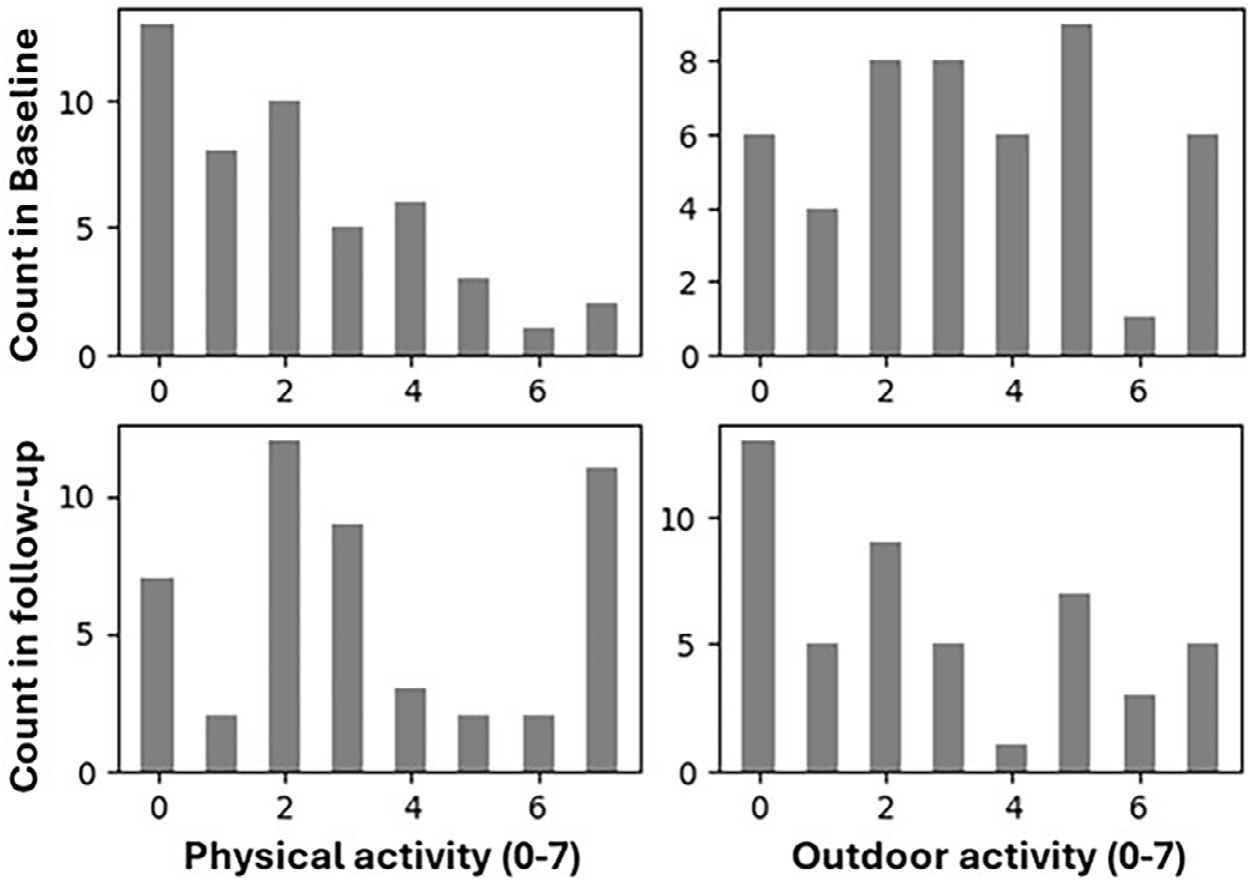
The distribution of physical activity and outdoor activity in baseline and final assessment.

For the 72 students who participated in the HWA research, the results indicated that physical activity levels increased along with time points (Figure 2). Mixed linear model regression results further revealed a significant increase for time effects on physical activity (*p*<0.001) (Table 1) but no time effects on outdoor activity (*p*>0.05). Posthoc analysis indicated that physical activity levels at time points 3 and 4 (3-year and 4-year follow-up assessments) are significantly higher than those at time points 0–1 (baseline and 1-year follow-up). However, there were no differences between time points 3 and 4, indicating that the improvement of physical activity reached a plateau and stop increasing.

**Table 1:** SVI Overall and Subscores and Ranking for U.S. Counties, Illinois Census Tracts, Census Tracts Where Study Participants Lived, and Census Tract Where Altus Academy Is Located

Geography	SES subtheme score	Household characteristics subtheme score	Racial and ethnic minority status subtheme score	Housing type and transportation subtheme score	Overall Social Vulnerability Index score
All U.S. counties (*n*=3,144)	2.4819 (0.5000)	2.4607 (0.5000)	0.4988 (0.4988)	2.4648 (0.5000)	7.9062 (0.5000)
All Illinois census tracts (*n*=3,263)	2.4888 (0.4999)	2.4605 (0.5001)	0.4993 (0.4992)	2.155 (0.5001)	7.604 (0.4999)
Illinois census tracts where participants lived (*n*=13)	2.9858 (0.6219)	2.3280 (0.4637)	0.5893 (0.5893)	2.2900 (0.5314)	8.1931 (0.5629)

**Figure 3:**
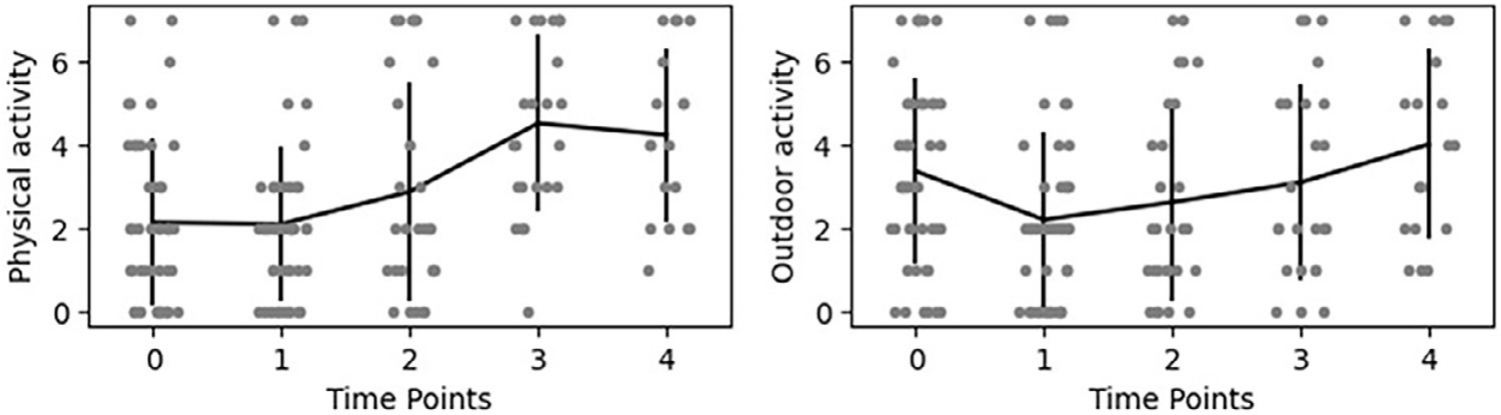
The changes in physical activity (left) and outdoor activity (right) along with data points.

Regarding the age effect, physical activity did not show a significant association with age at the baseline session (*p*=0.29) (Table 2). However, at the follow-up session, physical activity demonstrated a significant positive association with age (*p*=0.002), indicating that the physical activity level would be higher along with aging after participation in the HWA (Figure 3). Posthoc analysis did not find any significant difference between different ages, which might be attributed to the small sample size. For outdoor activity, there was no significant association with age at either the baseline or follow-up sessions, indicating consistency in outdoor activity across different age groups.

**Table 2:** The Effects of Age on Physical Activity and Outdoor Activity in Baseline and Follow-Up Data Points

Session	Variable	Coefficient	Z-value	*p-*value	Low CI	High CI
Baseline	Physical activity	0.13	1.05	0.29	−0.11	0.36
Baseline	Outdoor activity	−0.1	−1.08	−0.28	0.28	0.08
Follow-up	Physical activity	0.39	3.15	0.002	0.15	0.64
Follow-up	Outdoor activity	0.22	1.8	0.07	−0.02	0.45

## DISCUSSION

The HWA program was successfully implemented in Altus Academy over 7 years. Given the limited enrollment at Altus and that this was a pragmatic trial design, the inclusion of 48 participants was still remarkably encouraging. The results of this study indicate that the HWA program was able to effectively increase physical activity, and the improvement was related with the length of enrollment in the program. Furthermore, change in physical activity from baseline was also influenced by age of the participants because the adolescent group had a greater change. Finally, Altus Academy sits within the Lawndale neighborhood of Chicago, which is a historically underserved population and enrolls students who fall below the Illinois Self-Sufficiency Standards.^22^ Many participants live in areas that do not have access to outdoor parks and recreation nor provide safe walkable/playable areas, reflected by higher SVI scores in neighborhoods where students who attend Altus live. The fact that the HWA was able to improve self-reported physical activity in students from neighborhoods with higher social vulnerability, a factor known to negatively influences healthy lifestyle behaviors,^12^ is particularly important.

As part of the study aims, the effect of HWA on the amount of physical activity implemented by an HWA participant during a typical week was examined. As shown in Figure 2, Figure 3 and Table 3, a significant effect of years in the program on change in 60-minute physical activity was found. However, it is notable that the maximum change occurred from Years 3 to 4 with a plateau after that. This could be explained by maximum capacity to fit 60-minute physical activity into their weekly schedules on the basis of other extracurriculars that are offered as students get older. Similar to the findings of this study, others have shown that if interventions can be sustained over several years, there is a likelihood that the intervention is effective in producing behavior change and a sustained effort to be physically active.^23^^,^^24^ In addition, similar to HWA, when interventions are multicomponent and include educational components, they report higher efficacy.^24^^,^^25^ Of note, comparative results from this study and the existing literature are lacking because the results presented here examine weekly 60-minute physical activity over a longitudinal period in an underserved population. In addition, it must be noted that 30-minute outdoor physical activity did not show a significant change. This is not surprising given that many students report that outdoor space near their home is not conducive to exercise. Similar to the current findings, previous studies have shown that outdoor physical activity for children and adolescents can be limited by the condition of outdoor play space.^26^, ^27^, ^28^

**Table 3:** The Effects of Time Points on Physical Activity and Outdoor Activity

Variable	Independent	Coefficient	Z-value	*p*-value	Low CI	High CI
Physical activity	Time points	0.61	5.99	<0.001	0.41	0.81
Outdoor activity	Time points	0.082	0.731	0.47	−0.14	0.3

**Figure 4:**
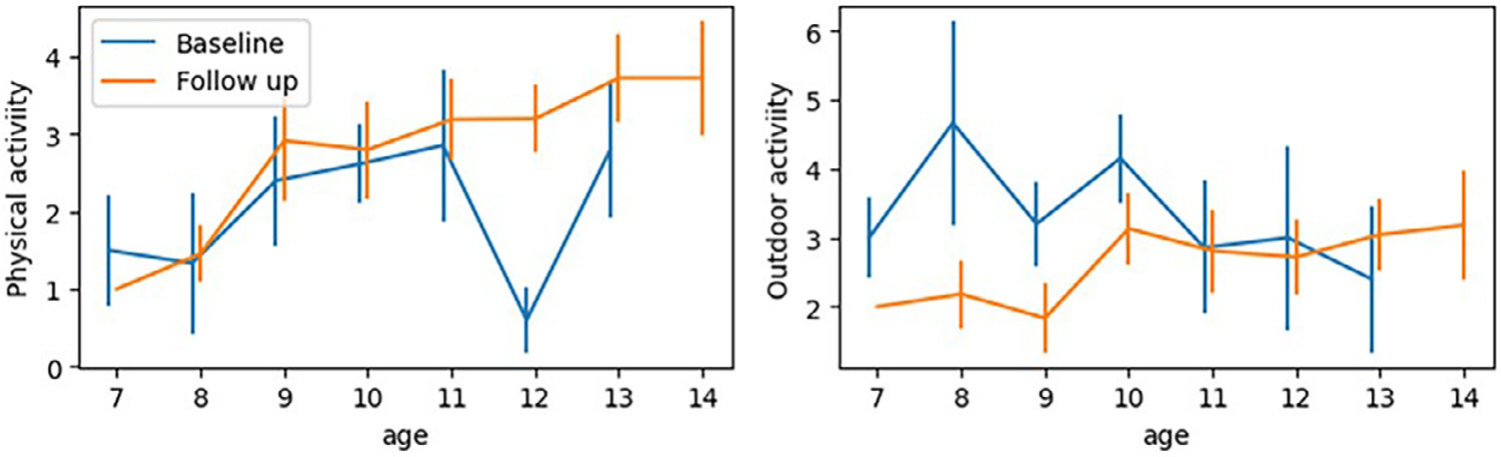
The changes in physical activity (left) and outdoor activity (right) along with age in baseline and follow-up assessments.

The current analysis also found that the age group of children differentially affected change in physical activity. In participants aged 7–14 years, their physical activity continued to increase as seen in Figure 4 and Table 2. These results are especially poignant when comparing with national and international trends in adolescent physical activity. Literature indicates that as children enter adolescence (aged approximately 8–10 years), physical activity begins to decline because there is an increase in sedentary behaviors.^27^, ^28^, ^29^, ^30^, ^31^ There are several factors cited for this phenomenon, including lack of participation in physical education programs and extracurriculars, increased screen time with increased academic work and social media use, and decreased self-esteem and self-efficacy as body composition changes.^18^, ^19^, ^20^^,^^29^^,^^30^ The positive impact of the HWA reported in this paper may be explained by their tailored approach to engaging adolescents in activities centered upon needs and interests. In this context, if there is a specific interest in athletics, lessons can be modified to fit those interests. In addition, the HWA works with a small group of middle schoolers, which allows for more one-on-one tailoring, with a specific focus on what motivates children aged 10–14 years to move their bodies more.

### Limitations

Altus Academy enrolls about 75–80 students in total from first through eighth grade per school year. Once students graduate or disenroll from Altus, they are also disenrolled from the HWA, and there are no further data collection or tracking on lifestyle behaviors. Because there can be high transiency within the school population, even though a participant may have consented, they will not have multiple data points because they have left before the end of the school year. In addition, there is no incentive for being a part of the study; therefore, parental consent can be inhibited. Finally, the self-reported survey tool relies on participants remembering what they had to eat the day before and how they exercise in any given week. This can be difficult for some participants. Future studies should focus on more refined data collection methods to demonstrate efficiency of comparable programs (e.g., accelerometry).

## CONCLUSIONS

Having a consistent and sustained intervention allows youth to be exposed to a variety of physical activity options. The tailored HWA model allows for youth to find ways to move more and sit less that are meaningful and relevant, facilitating implementation and prolonged adherence into an individual’s daily life. If health promotion programs are to positively impact physical activity levels in children and adolescents, there needs to be an emphasis on sustainability and feasibility. This allows youth to find personal relevance in the activity and connect to the movements in ways that will motivate them to continue the behavior as they get older. In addition, when paired with other healthy lifestyle habits, such as eating nutritious foods, physical activity interventions can be more consequential to improving the overall outlook on health and well-being. Feeling good about what you eat and how you move can go a long way.
